# Die Situation des deutschen Maßregelvollzugs – Ergebnisse einer Umfrage der DGPPN

**DOI:** 10.1007/s00115-023-01564-7

**Published:** 2023-11-09

**Authors:** Robert Zeidler, Manuela Dudeck, Udo Frank, Gabriel Gerlinger, Dirk Hesse, Jutta Muysers, Thomas Pollmächer, Christian Riedemann, Julia Sander, Birgit Völlm, Jürgen L. Müller

**Affiliations:** 1 Deutsche Gesellschaft für Psychiatrie und Psychotherapie, Psychosomatik und Nervenheilkunde e. V., Berlin, Deutschland; 2 Klinik für Forensische Psychiatrie und Psychotherapie der Universität Ulm am BKH Günzburg, Ulm, Deutschland; 3 ZfP Südwürttemberg, Ravensburg-Weissenau, Deutschland; 4 Maßregelvollzugszentrum Niedersachsen, Moringen, Deutschland; 5 LVR-Klinik Langenfeld, Langenfeld, Deutschland; 6 Zentrum für psychische Gesundheit, Klinikum Ingolstadt, Ingolstadt, Deutschland; 7 Maßregelvollzugszentrum Niedersachsen, Bad Rehburg, Deutschland; 8 Klinik für Forensische Psychiatrie, Zentrum für Nervenheilkunde, Universitätsmedizin Rostock, Rostock, Deutschland; 9 Klinik für Forensische Psychiatrie und Psychotherapie, Asklepios Fachklinikum Göttingen, Universitätsmedizin Göttingen, Rosdorfer Weg 70, 37081 Göttingen, Deutschland

**Keywords:** Forensische Psychiatrie und Psychotherapie, Straftäter, Überbelegung, Umfrage, Versorgung, Forensic psychiatry and psychotherapy, Health care, Offenders, Overcrowding, Survey

## Abstract

**Hintergrund und Fragestellung:**

Die Maßregeln nach den §§ 63 und 64 StGB wurden in der Vergangenheit wiederholt reformiert. Doch trotz der Novellierung des Rechts der Unterbringung (2016) mahnen Kliniken und Landesbehörden vor unzureichenden Kapazitäten und besorgniserregenden Zuständen. Die mediale Berichterstattung zeichnet ein herausforderndes Bild. Gleichzeitig mangelt es an validen Daten, die eine objektive Beschreibung der Situation im Maßregelvollzug (MRV) ermöglichen. Vor diesem Hintergrund wurden die Einrichtungsleitungen in Deutschland befragt.

**Material und Methoden:**

In dieser Onlineumfrage wurden 2021 alle 78 Einrichtungen des MRV in Deutschland zu Strukturdaten der Einrichtungen, zur Belegungs- und Personalsituation, zu besonderen Vorkommnissen, zur Unterstützung durch Fachaufsichten und Träger sowie zu besonderen Patientenmerkmalen befragt. Die Ergebnisse werden deskriptiv dargestellt.

**Ergebnisse:**

Von den 78 angeschriebenen Einrichtungen partizipierten 45 (58 %) an der Umfrage zumindest teilweise. Die Mehrzahl der Kliniken (68,5 %) beklagte eine deutliche Überbelegung. Es wurde ein deutlicher Mangel von Personal und Räumen berichtet, zugleich wurde angegeben, dass Patienten keine angemessene Behandlung erhalten. Etwa jeder 5. Patient war länger als 10 Jahre im MRV untergebracht. Jede 3. Klinik berichtete eine steigende Zahl an körperlichen Übergriffen durch Patienten.

**Diskussion:**

Der gewonnene Überblick zeigt die Kliniken des MRV in einer sehr unterschiedlichen, doch insgesamt angespannten Situation. Eine wesentliche Zahl der Kliniken steht unter großem Druck. Finanzielle, strukturelle, räumliche und personelle Ressourcen wurden als unzureichend beschrieben, den gesetzlichen Auftrag sach- und fachgerecht zu erfüllen. Die 2017 von der DGPPN vorgelegten Behandlungsstandards sind in vielen Kliniken nicht erfüllt.

**Zusatzmaterial online:**

Zusätzliche Informationen sind in der Onlineversion dieses Artikels (10.1007/s00115-023-01564-7) enthalten.

## Abstract (en)

**Background and objective:**

In German forensic psychiatry detention under Sections 63 and 64 of the German Penal Code have been repeatedly reformed over the past years; however, despite the most recent amendments to the law on detention, clinics and state authorities warn of insufficient capacities and worrying conditions. Media reports paint a defiant picture. At the same time, there is a lack of valid data that would allow an objective description of the situation in forensic psychiatry. Against this background the management of institutions in Germany has been surveyed.

**Material and methods:**

The survey was conducted as an online survey and sent to all 78 forensic hospitals in Germany. The survey covered topics such as structural data of the facilities, the occupancy and staffing situation, incidents, support from supervisory authorities and funding agencies, and patient characteristics. The results are presented descriptively.

**Results:**

Of the 78 facilities contacted, 45 (approximately 60%) participated at least partially in the survey. Many of the clinics (68.5%) complained of significant overcrowding. A clear lack of staff and rooms was reported, at the same time it was stated that patients do not receive adequate treatment. Approximately 1 in 5 patients have a length of stay for more than 10 years and one third of the clinics reported an increasing number of physical assaults by patients.

**Conclusion:**

This overview shows that the forensic psychiatric hospitals are in very different but generally strained situations. A significant number of clinics are under great pressure. Financial, structural, spatial and personnel resources were described as insufficient to properly and professionally fulfill the legal mandate. The treatment standards presented by the DGPPN in 2017 are not met in many clinics.

## Hintergrund

Die Maßregeln der Besserung und Sicherung sind eine im Strafrecht verankerte Sanktion für Straftäter, die in Folge einer überdauernden erheblichen psychischen Störung (§ 63 StGB) oder eines Hangs zur Einnahme psychotroper Substanzen (§ 64 StGB) weiterhin für gefährlich erachtet werden. Gegenwärtig sind etwa 8000 Patienten in einem Krankenhaus für forensische Psychiatrie, 4000 in einer Entziehungsanstalt untergebracht. Die Maßregeln wurden wiederholt reformiert, nicht zuletzt, um die Unterbringungsdauer und die Zuweisungszahlen zu begrenzen. Trotz der jüngsten Novellierung des Rechts der Unterbringung (2016) warnen Kliniken und Landesbehörden weiterhin vor unzureichenden Kapazitäten und vor besorgniserregenden Zuständen.

Die Anordnung der Maßregeln fällt in die Kompetenz des Bundes, deren Vollzug aber in die Regelungskompetenz der Bundesländer. Dementsprechend gibt es erhebliche Unterschiede, was die Zuweisungszahlen, die vorgehaltenen Betten, die für die Behandlung zur Verfügung gestellten Geld- und Personalmittel und die Unterbringungsdauern betrifft [1]. Insofern wiegen die Folgen des mit Freiheitsentzug verbundenen Grundrechtseingriffs für die Patientinnen und Patienten^1^ in den einzelnen Ländern unterschiedlich schwer [2].

Die Bedingungen im MRV stehen im kritischen Fokus der Öffentlichkeit, vor allem wegen Entweichungen oder Rückfalldelinquenz [3], obwohl diese insgesamt selten (unter 1 Entweichungsfall durch Flucht oder Ausbruch pro 100 Belegungsfälle pro Jahr) vorkommen [1]. Weniger offen werden Mängel der Qualität von Unterbringung und Behandlung diskutiert. 2021 wurde überregional über kritische Zustände innerhalb des Berliner Krankenhauses des Maßregelvollzugs (KMV) berichtet. 2020 wurden im KMV mehr als 300 gewalttätige Übergriffe auf Therapeuten – bis hin zur versuchten Tötung – dokumentiert; das KMV war bei dramatischer Überbelegung handlungsunfähig geworden und hatte keine Möglichkeit mehr, angemessen auf gefährliche Patienten zu reagieren [4].

Diese Berichte, auch aus anderen Bundesländern, sind im Wesentlichen nur anekdotisch. Verfügbare Datensätze bilden nicht alle Bundesländer ab und sind nicht öffentlich zugänglich [1, 5, 6], werden nicht regelmäßig publiziert (BAG; [7]) oder liegen bereits mehrere Jahre zurück.

Weitere Daten zu erheben, wurde wiederholt eingefordert [6]. Angesichts der offensichtlichen Notwendigkeit, zeitnah die Grundlage für eine datenfundierte Bewertung der Situation im MRV zu schaffen, führte eine Arbeitsgruppe der DGPPN eine einmalige Umfrage durch.

## Methodik

Die Autoren entwickelten einen Fragebogen mit 37 Items (s. Online-Supplement), mit dem zentrale Kennziffern des MRV nach §§ 63, 64 StGB im Zeitraum vom 07.09. bis 30.11.2021 erhoben wurden.

Grundlage der Angaben war die tagesaktuelle Situation in der jeweiligen Einrichtung, sofern nicht anders gefordert. Die Beantwortung der Umfrage erfolgte anonym und freiwillig. Alle Leiter und Leiterinnen der Einrichtungen des Maßregelvollzugs in Deutschland wurden per Mail kontaktiert und zur Teilnahme an der Onlineumfrage eingeladen.

Nach Abschluss der Onlinebefragung wurden die Daten aufbereitet und die Ergebnisse deskriptiv dargestellt.

Von den 78 angeschriebenen Einrichtungen nahmen 45 (58 %) an der Umfrage teil.

## Ergebnisse

### Strukturdaten

**Figure 1:**
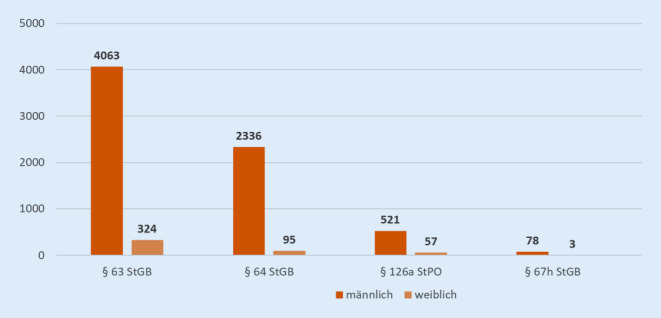

**Figure 2:**
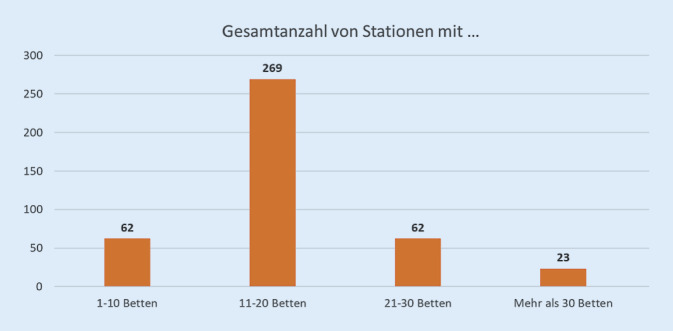

In den 45 befragten Einrichtungen waren zum Zeitpunkt der Datenerhebung insgesamt 7477 Patienten untergebracht. Davon waren 479 Frauen, was einem Anteil von 6,4 % entspricht (Abb. 1, 2).^2^

Elf Einrichtungen erklärten, dass jedes ihrer Patientenzimmer über eine Nasszelle verfügt. Im Median verfügten 56,6 % der Zimmer über eine Nasszelle mit einer weiten Spannweite von 2,6–93,7 %.

Einundvierzig von 42 Kliniken verfügten über Isolationsräume/Time-out-Räume, wobei Kliniken, die überwiegend Patienten gem. § 63 StGB betreuen, in etwa doppelt so viele Krisenräume vorhielten wie Entziehungsanstalten^3^.

**Figure 3:**
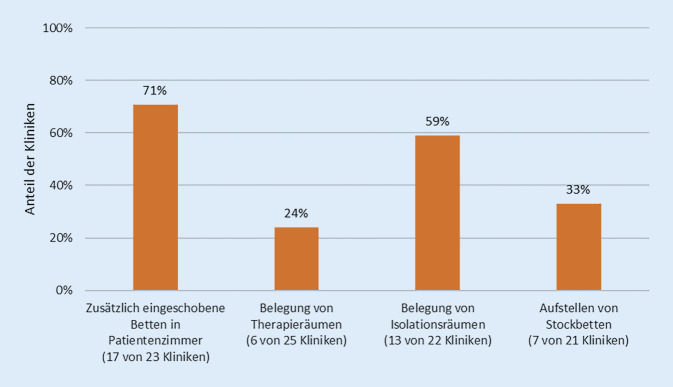

Etwa ein Drittel der Kliniken gab an, dass ihre Einrichtung überbelegt sei (Abb. 3).

**Figure 4:**
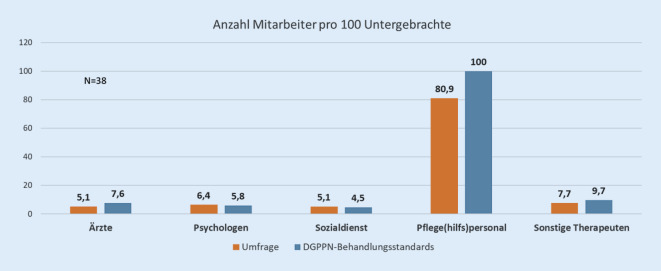

Anhand der Abb. 4 sieht man, dass die Empfehlungen zu Behandlungsstandards der DGPPN [9] nicht der Realität entsprechen.

Fast 60 % der Einrichtungen gaben an, dass erforderliche Therapieangebote aus finanziellen oder personellen Gründen nicht angeboten werden können.

### Besondere Vorkommnisse

#### Suizide

Für das Jahr 2019 wurden insgesamt 9 vollendete Suizide berichtet, das entspricht einem Anteil von 0,14 % (6450 Patienten in 38 Kliniken). In 5 Einrichtungen kam es zu jeweils einem, in 2 Einrichtungen zu je 2 Suiziden. Im Jahr 2020 wurden 11 vollendete Suizide verzeichnet (0,17 %), die sich auf 8 Einrichtungen verteilten; in 3 dieser Einrichtungen kam es zu jeweils 2 Suiziden.

#### Übergriffe

Körperliche Übergriffe auf Mitarbeitende und Mituntergebrachte zeigen die Tab. 1 und 2.

**Table 1:** 

	2019	2020
Einrichtungen mit körperlichen Übergriffen auf Mitarbeitende	27 von 35 (77 %)	27 von 37 (73 %)
Anzahl der Übergriffe auf Mitarbeitende pro Einrichtung/Jahr	17,9 (36,4)	19 (37,5)
*Anzahl der körperlichen Übergriffe auf ***Mitarbeitende*** pro Einrichtung/Jahr je 100 Untergebrachte*		
Kliniken gesamt	11,2 (21,7)	10,5 (18,2)
Kliniken mit > 80 % Patienten gemäß § 63 StGB	10,3 (16,2)	10,5 (18,3)
Kliniken mit > 80 % Patienten gemäß § 64 StGB	1,2 (2,6)	1,0 (1,9)
In bez. Rechtsgrundlage gemischteren Kliniken	15,9 (27,7)	15 (21,6)

*§ 63 StGB* Unterbringung in einem pschiatrischen Krankenhaus; *§ 64 StGB* Unterbringung in einer Entziehungsanstalt

**Table 2:** 

	2019	2020
Einrichtungen mit körperlichen Übergriffen auf Mituntergebrachte	29 von 33 (87,9 %)	34 von 36 (94,4 %)
Anzahl der Übergriffe auf Mituntergebrachte pro Einrichtung/Jahr	13,9 (15,9)	12,4 (14,3)
*Anzahl der körperlichen Übergriffe auf ***Mituntergebrachte*** pro Einrichtung/Jahr je 100 Untergebrachte*		
Kliniken gesamt	9,9 (10,6)	8,9 (9,5)
Kliniken mit > 80 % Patienten gemäß § 63 StGB	9,4 (9,8)	9,1 (10,4)
Kliniken mit > 80 % Patienten gemäß § 64 StGB	5,9 (10,7)	5,0 (7,6)
In bez. Rechtsgrundlage gemischteren Kliniken	11,8 (11,2)	10,3 (9,5)

*§ 63 StGB* Unterbringung in einem pschiatrischen Krankenhaus; *§ 64 StGB* Unterbringung in einer Entziehungsanstalt

**Figure 5:**
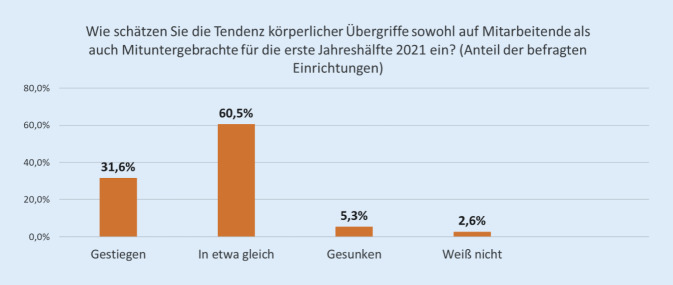

Etwa ein Drittel der Einrichtungen gab für die erste Jahreshälfte 2021 einen Anstieg von Übergriffen an (Abb. 5).

### Zwangsmaßnahmen

Als Zwangsmaßnahmen wurden die medikamentöse Zwangsbehandlung, die Fixierung, die Isolierung in einem speziell hierfür vorgesehenen Raum sowie der Zimmereinschluss, d. h. die Separierung von der Patientengruppe im eigenen Zimmer, erfasst. Die Häufigkeit ist in Tab. 3 zusammengefasst.

**Table 3:** 

	**Patienten nach § 63 untergebracht**	**Patienten nach § 64 untergebracht**	**Patienten nach § 126a untergebracht**
Medikamentöse Zwangsbehandlung	144/2971 = 4,8 % (*n* = 34)	1/2108 = 0,05 % (*n* = 36)	37/405 = 9,1 % (*n* = 36)
	**Anteil Patienten pro Einrichtung (gesamt)**	**In Einrichtungen mit >** **80** **% Patienten gemäß § 63**	
Fixierung	107/4684 = 2,3 % (*n* = 29)	36/1333 = 2,7 % (*n* = 11)	
Isolierung	950/4564 = 20 % (*n* = 29)	266/1160 = 22,9 % (*n* = 10)	
Zimmereinschluss	463/3255 = 14,2 % (*n* = 21)	56/713 = 7,8 % (*n* = 7)	

#### Dauerisolierte Patienten

Dauerisolierung wurde als Isolierung von mindestens einem Monat definiert. Insgesamt wurden 212 dauerisolierte Patienten genannt, wobei 20 Einrichtungen keine Dauerisolierungen berichteten. Dreizehn Institutionen hatten zwischen einem und 5 dauerisolierte Patienten, 4 eine 2‑stellige Anzahl an dauerisolierten Patienten in der Klinik.

#### Anzahl dauerfixierter Patienten

Auch hier wurde der Zeitrahmen als über einen Monat definiert. Es gab in der Stichprobe 4 dauerfixierte Patienten, die sich auf 3 Kliniken verteilten.

### Entlassungen

36 Kliniken mit 5556 Patienten berichteten von 638 Patienten, die zum Zeitpunkt der Befragung zusammenhängend länger als 10 Jahre in der jeweiligen Einrichtung waren. Das entspricht zwischen 0 und 39 % (*M* = 10,3 %) der Patienten pro Klinik. Für 48,1 % (307 von 638) von diesen Langzeituntergebrachten, sahen die Befragten auch in den nächsten fünf Jahren keine Entlassperspektive. In Kliniken, in denen mindestens 80 % der Patienten gemäß § 63 StGB untergebracht sind, lag der Anteil der bereits länger als zehn Jahre untergebrachten Patienten, zwischen 3,5 % und 39,7 % (*M* = 19,1 %) pro Klinik. Die drei wichtigsten Hinderungsgründe waren bleibende Gefährlichkeit, Therapieresistenz und fehlende Anschluss-Wohnform.

### Migrationshintergrund

Angaben zur Häufigkeit eines Migrationshintergrunds machten 35 Einrichtungen über insgesamt 5086 Patienten. Deren Anteil an der Gesamtbelegung variierte zwischen den Kliniken sehr, lag im Mittel bei 31,9 % (*n* = 1621) mit einer erheblichen Spannweite von 3,4–63,4 % und bei einem Süd-Nord- und West-Ost-Gefälle.

Von insgesamt 5594 Patienten hatten 424 (7,6 %) bei Aufnahme in den MRV keine ausreichenden Deutschkenntnisse, mit einer Spannweite von 0–25 % zwischen den Einrichtungen (*n* = 37).

### Unterstützung durch Fachaufsicht und Träger

Während 26 % der teilnehmenden Einrichtungen (*n* = 38) weder zufrieden noch unzufrieden mit der zuständigen Fachaufsicht waren, zeigte sich fast ein Drittel nicht mit der Unterstützung zufrieden. 3 % waren völlig unzufrieden. Demgegenüber waren 34 % der Einrichtungen zufrieden oder sogar voll zufrieden (8 %). Nahezu die Hälfte der Teilnehmenden (46 %) fühlte sich durch den Träger angemessen, 35 % jedoch nicht unterstützt (*n* = 37 Kliniken).

## Diskussion

Diese Umfrage wurde einmalig durchgeführt, um aktuelle relevante Kennzahlen über die Behandlung im MRV zu erheben. Es bildete sich die bundesweit sehr unterschiedliche Ausgestaltung des länderrechtlich geregelten MRV ab. Diese Beobachtung deckt sich auch mit der ebenfalls sehr heterogenen internationalen Datenlage [10]. Die Unterschiede betrafen z. B. Klinik- und Stationsgrößen, Unterbringungsdauer und Rahmenbedingungen, finanzielle und personelle Ausstattung.

### Strukturdaten

Die meisten Kliniken behandelten Patienten, die sowohl nach § 63 als auch des § 64 StGB untergebracht waren. Seit der Novellierung 2016 kam es in einzelnen Bundesländern vermehrt zu Zuweisungen im Rahmen einer einstweiligen Unterbringung gemäß § 126a StPO [6]. In dieser Umfrage waren es 578 Patienten (7,7 %). Bundesweit belastbare Zahlen liegen bislang nicht vor.

Die Einrichtungen waren in den einzelnen Bundesländern sehr heterogen ausgestattet. Etwa 700 Patienten waren bundesweit noch immer auf sehr großen Stationen mit 30 Betten und mehr untergebracht. Nur etwa zwei Drittel der Stationen entsprachen hinsichtlich der Größe den Empfehlungen der DGPPN von 2017 [9]. Dies erscheint sowohl unter therapeutischen als auch unter sicherheitsrelevanten Aspekten bedenklich. Nur rund die Hälfte der Kliniken verfügte über Einzelzimmer. Dies ist für die Versorgung der oft psychisch schwer gestörten und störungsbedingt gefährlichen Patienten nicht angemessen. Das Fehlen von Rückzugsmöglichkeiten allein führt zu vermehrten Konflikten. Der Zusammenhang zwischen räumlicher und personeller Ausstattung und Aggression in psychiatrischen Kliniken ist seit Jahren gut belegt [11].

Sowohl angesichts der oft jahrelangen Unterbringung als auch der potenziellen Bahnung von Zwischenfällen mit Gewalt gegen Mitbewohner sind Einzelzimmer zu fordern.

Angesichts der Überbelegung mussten Patienten in einigen Kliniken in Etagenbetten und/oder durch Belegung von Kriseninterventions‑, Therapie- und Versorgungsräumen untergebracht werden. Dies verschärft die Probleme durch die Überbelegung, führt zu „overcrowding“, behindert Therapie und nimmt der Klinik Handlungsmöglichkeiten im Krisenfall.

Die von den Kliniken berichteten Personalquoten unterschritten bei Ärzten, Pflege(hilfs)personal und Spezialtherapeuten die Empfehlungen der DGPPN 2017 (Abb. 4). So müssen zumindest Zweifel angemeldet werden, ob die Bundesländer ausreichend Ressourcen zur Verfügung stellen. Die Befragten beklagten, dass die Qualität der Behandlung bei einem Mangel an (Therapie‑)Räumen und fachlichem Personal leide und brachten vermehrte Zwangsmaßnahmen und eine Verlängerung der Unterbringung und damit auch einen Anstieg der Kosten in Zusammenhang mit der unzureichenden personellen und sächlichen Ausstattung.

### Besondere Vorkommnisse

Inhaftierungen und Unterbringungen gegen den Willen von Betroffenen stellen deutliche Risikofaktoren für Suizide dar [12, 13]. Männliche Inhaftierte haben eine 6fach höhere Suizidziffer gegenüber Männern in der Allgemeinbevölkerung, inhaftierte Frauen eine 9fach höhere Quote [14].

Nach erster Bewertung erscheinen die in der Umfrage mitgeteilten Suizidzahlen, bei allen methodischen Limitationen, auf einem leicht erhöhten Niveau z. B. zur Allgemeinpsychiatrie [15]. Angesichts der Unterschiede in Psychopathologie, Störungsbilder und deren Ausprägung, Komorbidität, Behandlungssetting und -dauer sowie der Rahmenbedingungen der Unterbringung sind diese Zahlen allenfalls sehr eingeschränkt mit denen der Allgemeinbevölkerung oder denen in allgemeinpsychiatrischen Behandlungssettings oder im Justizvollzug zu vergleichen. Eine systematische bundesweite Erhebung ist dringend zu fordern.

Die Ergebnisqualität im MRV wird auch durch Übergriffe gegen Mitarbeitende und Mituntergebrachte und Delikte während der Unterbringung gekennzeichnet [9]. Aggressive Übergriffe werden zumindest in Einzelfällen in Zusammenhang mit Missständen in den Kliniken gebracht. Es zeigen sich bei der Zahl der Übergriffe deutliche Unterschiede zwischen den Kliniken. Die Ergebnisse dieser Umfrage weisen im Jahr, über alle Einrichtungen gemittelt, 10 bis 11 körperliche Übergriffe auf Mitarbeiter je 100 Patienten aus. Zusätzlich kommt es zu 9 bis 10 Übergriffen auf Mituntergebrachte pro 100 Patienten. Die Häufigkeit aggressiver Vorfälle ist aus methodischen Gründen nur eingeschränkt in die Literatur einzuordnen. Neben Patientenmerkmalen sind auch Faktoren wie Stationsklima, quantitative und qualitative personelle Ausstattung [16], Architektur und das Raumangebot maßgeblich [17].

### Zwangsmaßnahmen

Zwangsmaßnahmen sind die Ultima Ratio im Umgang mit aggressivem und herausforderndem Verhalten. Sie bedeuten für die Betroffenen einen massiven Einschnitt in ihre Autonomie, können zu (Re‑)Traumatisierungen führen und belasten das Verhältnis zwischen Patienten und Behandlern. Zwangsmaßnahmen dürfen daher nur unter strikt definierten Bedingungen durchgeführt werden. Trotzdem ist etwa ein Drittel der Patienten, die gegen ihren Willen untergebracht sind, sowohl in der Allgemeinpsychiatrie [18] als auch in der forensischen Psychiatrie [19] von einer Zwangsmaßnahme betroffen.

Verschiedene Studien zeigen eine starke Varianz sowohl in der Art der angewandten Zwangsmaßnahmen als auch ihrer Prävalenz und Dauer. In der vorliegenden Erhebung waren innerhalb eines Jahres jeder 5. der Untergebrachten von einer Isolierung, 2 % von einer Fixierung und etwa 3 % von einer medikamentösen Zwangsmaßnahme betroffen. Einzelne Patienten im Maßregelvollzug mussten über lange Zeiträume hinweg isoliert oder gar fixiert werden. Diese Ergebnisse ähneln bei eingeschränkter Vergleichbarkeit den in der Literatur gefundenen Studien. Die Prävalenz von Isolierungen bei forensischen Patienten wird in anderen Studien mit 22,6 % ähnlich hoch beschrieben und ist damit deutlich häufiger als bei nichtforensischen Patienten (2,9 %; [20]). Für Fixierungen finden andere Studien mit 3,8 % ebenfalls eine ähnlich hohe Prävalenz bei forensischen Patienten [20]. Bei nichtforensischen Patienten ist die Prävalenz von Fixierungen im Vergleich etwas höher (4,7 %; [20]). Die Anwendung medikamentöser Zwangsbehandlungen wird bezogen auf die Allgemeinpsychiatrie seltener berichtet (0,4–0,6 % vs. 3 %; [20, 21]).

Jede einzelne dieser Maßnahmen ist, unabhängig von ihrer Dauer, zu viel und muss vermieden werden. Die Heterogenität der Häufigkeit der Maßnahmen muss Anlass sein, die Hintergründe aufzuklären und Verbesserungspotenziale zu identifizieren.

### Entlassungen

Die Ergebnisse der vorliegenden Befragung tragen der Heterogenität der länderrechtlich geregelten Gestaltung der Einrichtungen, den Unterschieden zwischen den Störungsbildern, deren Behandelbarkeit und deren Verlauf Rechnung. Es fließen aber auch die Urteilspraxis und Gepflogenheiten der zuständigen Gerichte, die Verfügbarkeit komplementärer Einrichtungen und von Anschlussbetreuungsangeboten mit ein. Die Unterbringungsdauern variieren zwischen den Ländern erheblich (z. B. 2019 Dauer bei Beendigung der Maßregel nach § 63 StGB 81,3 Monate [6,7 Jahre] bis 153,4 Monate [12,8 Jahre]; [1]).

Die drei wichtigsten Entlasshindernisse waren bleibende Gefährlichkeit, Therapieresistenz und eine fehlende Möglichkeit des Anschlusswohnens. Insbesondere fehlende Entlassmöglichkeiten sind ein vermeidbares Hindernis und führen zu unverhältnismäßiger Verlängerung des strafrechtlichen Freiheitsentzugs.

### Migrationshintergrund

Der Maßregelvollzug hat in den vergangenen 10 Jahren eine deutliche Internationalisierung erfahren, sodass der Anteil der Untergebrachten mit Migrationshintergrund aktuell bei ca. 30 % liegt. Die Probleme liegen hier nicht nur in der Sprachbarriere, der mittlerweile über das Modellprojekt SPRINT in Hessen hinaus Rechnung getragen wird, sondern insbesondere in der transkulturellen Herausforderung, dass viele Nationalitäten auf engsten Raum mit unterschiedlicher Sozialisierung unter Verlust ihrer Autonomie unter Zwang therapiert werden müssen [22]. Für einige dieser Personen besteht bereits vor Aufnahme in den Maßregelvollzug ein Abschiebebefehl, der die Therapie im Maßregelvollzug konterkariert. Das Erlernen von Deutsch hilft bei der Resozialisierung im Ursprungsland nicht. Die Resozialisierung in Deutschland stößt auf vielfältige Herausforderungen. Oftmals dient die Aufnahme in den Maßregelvollzug überwiegend der Sicherung psychisch und suchtkranker Straftäter. Diese Ergebnisse stützen die Forderung nach therapieorientierten Lösungen.

## Limitationen

Es handelt sich um eine einmalige Erhebung. Wenngleich die Rücklaufquote sehr gut war und die Mehrheit von Patienten und Einrichtungen repräsentiert wurde, kann die fast 40 %ige Nonresponderrate das Bild verzerren. Außerdem beruhen die Angaben zum Teil auf subjektiven Einschätzungen der Befragten.

## Fazit

Der gewonnene Überblick zeigt die Kliniken in sehr unterschiedlichen, doch insgesamt angespannten Lagen. Eine wesentliche Zahl der Klinikleiter sieht die Kliniken unter großem Druck, bei unzureichenden finanziellen, strukturellen, räumlichen oder personellen Ressourcen den gesetzlichen Auftrag sach- und fachgerecht zu erfüllen. Der Belegungsdruck verstärkt nicht angemessene Unterbringungsbedingungen durch Überbelegung und Personalknappheit weiter. Die 2017 von der DGPPN vorgelegten Behandlungsstandards sind in vielen Kliniken nicht erfüllt. Angesichts der grundrechtsrelevanten Eingriffe im Rahmen der Maßregeln muss eine einheitliche und auskömmliche Ausstattung sichergestellt sein.

### Supplementary Information






## Footnotes

1 Aufgrund der besseren Lesbarkeit und der Platzlimitation wird im Folgenden der Begriff Patienten verwendet.

2 2019 waren bundesweit insgesamt 12.343 Personen untergebracht [8]. Neuere Zahlen liegen nicht vor.

3 Bezogen jeweils auf Kliniken, die mindestens zu 80 % mit Patienten gem. §§ 63 oder 64 StGB belegt sind.
