# Erratum zu: Die Situation des deutschen Maßregelvollzugs – Ergebnisse einer Umfrage der DGPPN

**DOI:** 10.1007/s00115-023-01582-5

**Published:** 2024-01-04

**Authors:** Robert Zeidler, Manuela Dudeck, Udo Frank, Gabriel Gerlinger, Dirk Hesse, Jutta Muysers, Thomas Pollmächer, Christian Riedemann, Julia Sander, Birgit Völlm, Jürgen L. Müller

**Affiliations:** 1 Deutsche Gesellschaft für Psychiatrie und Psychotherapie, Psychosomatik und Nervenheilkunde e. V., Berlin, Deutschland; 2 Klinik für Forensische Psychiatrie und Psychotherapie der Universität Ulm am BKH Günzburg, Ulm, Deutschland; 3 ZfP Südwürttemberg, Ravensburg-Weissenau, Deutschland; 4 Maßregelvollzugszentrum Niedersachsen, Moringen, Deutschland; 5 LVR-Klinik Langenfeld, Langenfeld, Deutschland; 6 Zentrum für psychische Gesundheit, Klinikum Ingolstadt, Ingolstadt, Deutschland; 7 Maßregelvollzugszentrum Niedersachsen, Bad Rehburg, Deutschland; 8 Klinik für Forensische Psychiatrie, Zentrum für Nervenheilkunde, Universitätsmedizin Rostock, Rostock, Deutschland; 9 Klinik für Forensische Psychiatrie und Psychotherapie, Asklepios Fachklinikum Göttingen, Universitätsmedizin Göttingen, Rosdorfer Weg 70, 37081 Göttingen, Deutschland

**Erratum zu:**

**Nervenarzt 2023**

10.1007/s00115-023-01564-7

In diesem Artikel wurde der Name der Autorin Jutta Muysers fälschlicherweise als Jutta Muyers angegeben.

Der Originalbeitrag wurde korrigiert.

