# Supplementary material for: The situation in the German forensic commitment—Results of a survey by the DGPPN
Source: Nervenarzt. 2023 Nov 9;95(1):1–8. [Article in German] doi: 10.1007/s00115-023-01564-7 (PMC10810040; doi:10.1007/s00115-023-01564-7)
Supplement: Supplementary file 1 [file 115_2023_1564_MOESM1_ESM.docx]

# Online-Supplements

**eTabelle 1:** Übergriffe auf Mitarbeiter, N = Anzahl der Einrichtungen, MW = Mittelwert, SD = Standardabweichung, Min. = Minimum, Max. = Maximum

| ​ | | 2019 | 2020 |
| --- | --- | --- | --- |
| Einrichtungen mit körperlichen Übergriffen auf Mitarbeiter | | 27 von 35 (77 %) | 27 von 37 (73 %) |
| Anzahl der Übergriffe auf Mitarbeiter pro Einrichtung/ Jahr | | MW = 17,9, SD = 36,4,  Min. = 0  Median = 7  Max. = 200 | MW = 19, SD = 37,5  Min. = 0  Median = 5  Max. = 159 |
| Anzahl der körperlichen Übergriffe auf **Mitarbeiter** pro Einrichtung/ Jahr, je 100 Untergebrachte | | | |
|  | Kliniken gesamt | N = 35, MW = 11,2, SD = 21,7  Min. = 0  Median = 4,3  Max. = 117 | N = 37, MW = 10,5, SD = 18,2  Min. = 0  Median = 4,3  Max.= 88,9 |
|  | Kliniken mit >80 % Pat. gemäß § 63 StGB | N = 11, MW = 10,3, SD = 16,2  Min. = 0  Median = 3,1  Max. = 43,5 | N= 12, MW = 10,5, SD = 18,3  Min. = 0  Median = 2,7  Max.= 59,3 |
|  | Kliniken mit >80 % Pat. gemäß § 64 StGB | N = 7, MW = 1,2, SD = 2,6  Min. = 0  Median = 0  Max. = 7,1 | N = 8, MW = 1,0, SD = 1,9  Min. = 0  Median = 0  Max.= 4,7 |
|  | in bzgl. Rechtsgrundlage gemischteren Kliniken | N = 17, MW = 15,9, SD = 27,7  Min. = 0  Median = 5,4  Min = 0, Max. = 117 | N = 17, MW = 15, SD = 21,6  Min. = 0  Median = 6,6  Min. = 0, Max. = 88,9 |

**eTabelle 2:** Übergriffe auf Mit-Untergebrachte, N = Anzahl der Einrichtungen, MW = Mittelwert, SD = Standardabweichung, Min. = Minimum, Max. = Maximum

| ​ | | 2019 | 2020 |
| --- | --- | --- | --- |
| Einrichtungen mit körperlichen Übergriffen auf Mit-Untergebrachte | | 29 von 33 (87,9 %) | 34 von 36 (94,4 %) |
| Anzahl der Übergriffe auf Mit-Untergebrachte pro Einrichtung/ Jahr | | N= 33, MW = 13,9, SD = 15,9  Min. = 0  Median = 7  Max. = 62 | MW = 12,4, SD= 14,3,  Min. = 0  Median = 6  Max. = 58 |
| Anzahl der körperlichen Übergriffe auf **Mit-Untergebrachte**  pro Einrichtung/ Jahr, je 100 Untergebrachte | | | |
|  | Kliniken gesamt | N = 33, MW = 9,9, SD = 10,6  Min. = 0  Median = 5,5  Max. = 36,3 | N = 36, MW = 8,9, SD = 9,5  Min. = 1,54  Median = 6  Max. = 39 |
|  | Kliniken mit >80 % Pat. gemäß § 63 StGB | N = 11, MW = 9,4, SD = 9,8  Min. = 0  Median = 8,6  Max. = 33,8 | N = 12, MW = 9,1, SD = 10,4 Min. = 1,5,  Median = 7,6  Max. = 39,3 |
|  | Kliniken mit >80 % Pat. gemäß § 64 StGB | N = 6, MW = 5,9, SD = 10,7,  Min. = 0  Median = 2,3  Max. = 27,48 | N = 7, MW = 5,0, SD = 7,6  Min. = 0  Median = 2,5  Max. = 21,4 |
|  | in bzgl. Rechtsgrundlage gemischteren Kliniken | N = 16, MW = 11,8, SD = 11,2  Min. = 1  Median = 7,7  Max. = 36,3 | N = 17, MW = 10,3, SD = 9,5  Min. = 0  Median = 8,1  Max. = 33,9 |
