# Supplementary material for: The situation in the German forensic commitment—Results of a survey by the DGPPN
Source: Nervenarzt. 2023 Nov 9;95(1):1–8. [Article in German] doi: 10.1007/s00115-023-01564-7 (PMC10810040; doi:10.1007/s00115-023-01564-7)
Supplement: Supplementary file 2 [file 115_2023_1564_MOESM2_ESM.pdf]

## Allgemeine Angaben

1. In welchem Bundesland liegt Ihre Einrichtung?

## Strukturdaten

### 2. Wie viele PatientInnen in Ihrer Einrichtung sind

**Nach § 63 StGB untergebracht?**

Wie viele davon sind Frauen?

Wie viele davon sind divers?

**Nach § 64 StGB untergebracht?**

Wie viele davon sind Frauen?

Wie viele davon sind divers?

**Nach § 126a StPO untergebracht?**

Wie viele davon sind Frauen?

Wie viele davon sind divers?

**Nach § 67h StGB untergebracht?**

Wie viele davon sind Frauen?

Wie viele davon sind divers?

### 3. Wie hoch ist die Anzahl von Stationen in Ihrer Einrichtung mit einer Belegung von ...

1-10 Betten

11-20 Betten

21-30 Betten

Über 30 Betten

4. Welche Zimmer sind in welcher Anzahl in Ihrer Einrichtung vorhanden?

Anzahl Einbettzimmer

Anzahl Zweibettzimmer

Anzahl Dreibettzimmer

Anzahl Zimmer für mehr als drei Personen

5. Wie viele PatientInnenzimmer in Ihrer Einrichtung verfügen über eine eigene Nasszelle? (Angabe in absoluten Zahlen)

6. Wie hoch ist die Anzahl der Isolationszimmer/Time-Out-Räume?

## Belegungs- und Personalsituation

7. Ist Ihre Einrichtung derzeit überbelegt?

- ○ Ja
- ○ Nein

Kommentare/Gründe

[ ]

8. Wie kompensieren Sie die Überbelegung? (Anzahl Betten angeben)

Belegung von Therapieräumen [ ]

Belegung von Isolations-/ Time-Out-Räumen [ ]

Zusätzlich eingeschobene Betten in Patientenzimmern [ ]

Aufstellen von Stockbetten [ ]

Sonstiges [ ]

9. Gibt es in Ihrer Einrichtung Nachteinschluss?

- ○ Ja
- ○ Nein

10. Wie sehr stimmen Sie der Aussage zu: "Die verfügbaren Räumlichkeiten in unserer Einrichtung sind zur Erreichung unserer Aufgaben ausreichend."

- ○ Stimme überhaupt nicht zu
- ○ Stimme nicht zu
- ○ Stimme weder zu noch lehne ich ab
- ○ Stimme zu
- ○ Stimme voll und ganz zu

Hier können Sie Ihre Aussage begründen.

[ ]

11. Wieviel Personal (in Vollkräften) der folgenden Berufsgruppen steht Ihnen tatsächlich zur Verfügung?

ÄrztInnen

PsychologInnen

SozialarbeiterInnen

Pflegepersonal

Pflegehilfspersonal

SpezialtherapeutInnen (z. B. Ergotherapie, Kunsttherapie)

Sicherheitsdienst

12. Wie viele Personalstellen sind bei Ihnen derzeit unbesetzt?

ÄrztInnen

PsychologInnen

SozialarbeiterInnen

Pflegepersonal

Pflegehilfspersonal

SpezialtherapeutInnen (z. B. Ergotherapie, Kunsttherapie)

Sicherheitsdienst

13. Wie hoch war die prozentuale Personal-Ausfallzeit bei Ihnen im Jahr 2020?

Gesamtausfallzeit (Krankenstand + Urlaub, Fortbildungen etc.)

Nur Krankenstand

14. Haben Sie in Ihrer Einrichtung Personalanhaltszahlen oder ein Budget?

- ◯ Personalanhaltszahlen
- ◯ Budget

15. Wie sehr stimmen Sie der Aussage zu: "Die verfügbaren Personalressourcen in unserer Einrichtung sind zur Erreichung unserer Aufgaben ausreichend."

- ◯ Stimme überhaupt nicht zu
- ◯ Stimme nicht zu
- ◯ Stimme weder zu noch lehne ich ab
- ◯ Stimme zu
- ◯ Stimme voll und ganz zu

Hier können Sie Ihre Aussage begründen.

## Besondere Vorkommnisse

16. Wie viele vollendete Suizide gab es in Ihrer Einrichtung?

Im Jahr 2019

Im Jahr 2020

17. Wie viele körperliche Übergriffe gegenüber Personal wurden in Ihrer Einrichtung erfasst/dokumentiert.

Im Jahr 2019

Im Jahr 2020

18. Wie viele körperliche Übergriffe gegenüber MitpatientInnen wurden in Ihrer Einrichtung erfasst/dokumentiert.

Im Jahr 2019

Im Jahr 2020

19. Wie schätzen Sie die Tendenz der körperlichen Übergriffe für die erste Jahreshälfte 2021 ein?

○ Gestiegen ○ In etwa gleich ○ Gesunken ○ Weiß nicht

20. Hat sich die Anzahl dieser Vorfälle in den letzten Jahren verändert?

○ Ja

○ Nein

Erläuterungen/Gründe

21. Hat sich die Qualität dieser Vorfälle in den letzten Jahren verändert?

○ Ja

○ Nein

Erläuterungen/Gründe

22. Bei wie vielen PatientInnen wurden in Ihrer Einrichtung im Jahr 2020 Zwangsbehandlungen (medikamentös) durchgeführt?

Bei PatientInnen, die nach § 63 StGB untergebracht sind

Bei PatientInnen, die nach § 64 StGB untergebracht sind

Bei PatientInnen, nach § 126a StGB untergebracht sind

23. Wie oft kam es in Ihrer Einrichtung im Jahr 2020 zu einer räumlichen Absonderung im eigenen Zimmer (Zimmereinschluß)?

Anzahl der Maßnahme

Anzahl der PatientInnen, bei denen die Maßnahme zur Anwendung kam

Durchschnittliche Dauer der Maßnahme

24. Wie oft kam es in Ihrer Einrichtung im Jahr 2020 zu einer Isolierung (in besonders gesichertem Raum mit nicht regulärer Möblierung)?

Anzahl der Maßnahme

Anzahl der PatientInnen, bei denen die Maßnahme zur Anwendung kam

Durchschnittliche Dauer der Maßnahme

25. Wie oft kam es in Ihrer Einrichtung im Jahr 2020 zu einer Fixierung?

Anzahl der Maßnahme

Anzahl der PatientInnen, bei denen die Maßnahme zur Anwendung kam

Durchschnittliche Dauer der Maßnahme

26. Wie viele dauerisolierte PatientInnen (Isolierung mind. 1 Monat) waren im Jahr 2020 in Ihrer Einrichtung zu versorgen?

27. Wie viele dauerfixierte PatientInnen (Fixierung mind. 1 Monat) waren im Jahr 2020 in Ihrer Einrichtung zu versorgen?

## Unterstützung

28. Wie zufrieden sind Sie mit Unterstützung von Seiten der Fachaufsicht (z. B. bei der Personalsuche, bei Überbelegung o. ä.)?

- ◯ Überhaupt nicht zufrieden
- ◯ Nicht zufrieden
- ◯ Weder zufrieden noch unzufrieden
- ◯ Zufrieden
- ◯ Voll und ganz zufrieden

29. Wie zufrieden sind Sie mit Unterstützung von Seiten des Trägers (z. B. bei der Personalsuche, bei Überbelegung o. ä.)?

- ◯ Überhaupt nicht zufrieden
- ◯ Nicht zufrieden
- ◯ Weder zufrieden noch unzufrieden
- ◯ Zufrieden
- ◯ Voll und ganz zufrieden

## Besondere Patientenmerkmale

30. Wie viele Ihrer PatientInnen haben eine eigene Migrationserfahrung? (Angabe in absoluten Zahlen)

[ ]

31. Wie viele PatientInnen haben bei Aufnahme aus Ihrer Sicht keine ausreichenden Deutschkenntnisse für eine regelhafte Behandlung im Maßregelvollzug? (Angabe in absoluten Zahlen)

[ ]

32. Wie viele PatientInnen haben Sie, die zusammenhängend länger als 10 Jahre in der Unterbringung sind? (Angabe in absoluten Zahlen)

[ ]

33. Bei wie vielen von diesen PatientInnen sehen Sie keine Entlassperspektive in den nächsten fünf Jahren? (Angabe in absoluten Zahlen)

[ ]

34. Was sind die drei wichtigsten Hinderungsgründe für PatientInnenentlassungen?

- ☐ Keine Anschlusswohnform
- ☐ Therapieresistenz
- ☐ Therapieverweigerung
- ☐ Fehlende Finanzierung der Nachbetreuung
- ☐ Lockerungsmissbrauch
- ☐ Übergriffe
- ☐ Drogenrückfälle
- ☐ Gefährlichkeit
- ☐ Ausländerrechtliche Gründe
- ☐ Kein stabilisierendes soziales Entlassfeld
- ☐ Fehlende Kooperationsbereitschaft des Untergebrachten für eine Entlassvorbereitung (z. B. fehlende Bereitschaft zum Einsatz von Eigenmitteln, Hospitalisierung)

35. Wie viele Verhältnismäßigkeitsentlassungen (Erledigungen) hatten Sie im Jahr 2020?

Bei PatientInnen nach § 63 StGB [ ]

Bei PatientInnen nach § 64 StGB [ ]

36. Können erforderliche Therapieangebote aus finanziellen oder personellen Gründen nicht angeboten werden?

- ◯ Ja
- ◯ Nein

Erläuterungen/Gründe

## Abschlussfrage

37. Im Feld sind weitere Umfragen geplant, z. B. eine Stichtagserhebung. Wie ist Ihre Meinung dazu?

| | Sehr wichtig | Wichtig | Nicht so wichtig | Unwichtig |
|---|---|---|---|---|
| Ich finde eine solche Erhebung | ○ | ○ | ○ | ○ |
